# How an electronic health record became a real-world research resource: comparison between London’s Whole Systems Integrated Care database and the Clinical Practice Research Datalink

**DOI:** 10.1186/s12911-020-1082-7

**Published:** 2020-04-20

**Authors:** Alex Bottle, Carole Cohen, Amanda Lucas, Kavitha Saravanakumar, Zia Ul-Haq, Wayne Smith, Azeem Majeed, Paul Aylin

**Affiliations:** 1 Dr Foster Unit, Imperial College London, School of Public Health, Imperial College London, 3 Dorset Rise, London, EC4Y 8EN UK; 2 Imperial College Health Partners, London, UK

**Keywords:** Data warehousing, Electronic medical records, Integrated care, Real world evidence, Clinical practice research Datalink

## Abstract

**Background:**

In the UK, several initiatives have resulted in the creation of local data warehouses of electronic patient records. Originally developed for commissioning and direct patient care, they are potentially useful for research, but little is known about them outside their home area. We describe one such local warehouse, the Whole Systems Integrated Care (WSIC) database in NW London, and its potential for research as the “Discover” platform. We compare Discover with the Clinical Practice Research Datalink (CPRD), a popular UK research database also based on linked primary care records.

**Methods:**

We describe the key features of the Discover database, including scope, architecture and governance; descriptive analyses compare the population demographics and chronic disease prevalences with those in CPRD.

**Results:**

As of June 2019, Discover held records for a total of 2.3 million currently registered patients, or 95% of the NW London population; CPRD held records for over 11 million. The Discover population matches the overall age-sex distribution of the UK and CPRD but is more ethnically diverse. Most Discover chronic disease prevalences were comparable to the national rates. Unlike CPRD, Discover has identifiable care organisations and postcodes, allowing mapping and linkage to healthcare provider variables such as staffing, and includes contacts with social, community and mental health care. Discover also includes a consent-to-contact register of over 3000 volunteers to date for prospective studies.

**Conclusions:**

Like CPRD, Discover has been a number of years in the making, is a valuable research tool, and can serve as a model for other areas developing similar data warehouses.

## Background

The healthcare sector and regulatory bodies increasingly need to understand more about the real-world implications of diseases and healthcare interventions, requiring access to good-quality fully integrated healthcare datasets. England’s National Health Service (NHS) is well placed to deliver this for several reasons. With a single healthcare system, it is possible to follow patients from birth to death. With a low proportion of healthcare services being provided outside of the NHS (£9bn compared with £126bn in the NHS in 2017), [1] it is possible to obtain a near-complete view of both existing and new services and treatments that patients access. The computerisation of UK general practice records and the fact that 98% of the population is registered with a GP leads to almost whole-population coverage. Unlike with clinical trials and biobanks, the denominator resulting from such databases is relatively free from selection bias and represents the entire population.

While primary care sees most contacts with patients, linkage with secondary care and other sectors is needed for a full picture of the patient’s journey through the health and social care system and their outcomes. In England, as in the other UK countries, hospital inpatient data are combined to give a single database, but primary care uses several different systems, so far preventing the creation of an equivalent database for primary care. Instead, large samples of vendor-specific primary care data for research are available from several sources. The Clinical Practice Research Datalink (CPRD), for example, now includes records of over 11 million currently registered patients (16% of UK population), with linkage to hospital records, the national cancer registry, area-level social deprivation information and national mortality data, though some of these sources are for England only; the Health Improvement Network (THIN) [2] and QResearch [3] databases are similar but smaller. CPRD has generated over 1000 research papers [4]. Various initiatives have created local data warehouses such as the KID in Kent [5]. It uses pseudonymisation-at-source to link patient-level records from services including general practices, hospitals, community health services, hospices, and adult social care for its nearly two million population in SE England. It was established to track service use by patients with any of a set of long-term conditions but has since expanded to cover all patients. It is overseen by a steering group, one of whose subgroups considers requests for access to the data. These are not epidemiological cohorts or resources like UK Biobank [6] but were developed primarily for direct patient care and commissioning. Technical issues such as interoperability of data systems and ethics have complicated their construction.

Over the past 5 years, the team behind one such local data warehouse in North West London has overcome such issues to make the dataset available for research. We cover the origin, funding, contents and structure of this data warehouse, derived from the Whole System Integrated Care (WSIC) programme, its anonymised research version Discover, and its consent-to-contact feature. We then compare it with CPRD and discuss access to Discover and its current and future uses and developments for research.

### Origins and uses of the WSIC database

Commissioning is the process by which health and care services are planned, purchased and monitored. Within the NHS, local Clinical Commissioning Groups (CCGs) are responsible for planning, designing, buying, and paying for most NHS services including urgent and emergency care, acute care, mental health and community services across England (the commissioning landscape is changing: see [7] for a review). The need for a data warehouse was identified during a programme of consultation on the journey towards integrated care led by eight CCGs in North West London. As in many healthcare systems, medical records are held in database silos, and the need to share information about how patients go through healthcare organisations was recognised as a critical success factor.

The initial requirement for information sharing was to improve patient care, including by developing analytics to prioritise patients who may benefit from proactive intervention e.g. through risk stratification [8]. Whole-system activity was used to calculate a patient system cost. There was also a need for population-based data to inform the development of what was known as “accountable care partnerships”, in which healthcare providers work with a single pooled budget to take joint responsibility for delivering services for a defined population. The WSIC dataset was therefore created, covering primary care, community and mental health care, secondary and tertiary care, emergency departments and social care.

The WSIC database is currently used for direct patient care, service evaluation, commissioning and now also for research as Discover. For direct patient care, the WSIC team developed disease-specific dashboards, which can be accessed by healthcare professionals with a legitimate relationship with WSIC. For other uses, the database is de-identified. The challenges for using linked databases for service evaluation include data quality (coverage, completeness and accuracy) and producing actionable information from the data. For example, evaluating whether a new service reaches the target population better than the old model requires sufficient years of comparable data before and after the change. It also requires appropriate denominator data, i.e. the whole target population and not just those who actually use the service and are thereby captured electronically. Capturing clinical processes in hospital for audit is still usually done using purpose-built audit databases, as administrative data are very limited in what process measures can be constructed from them. Ideally, processes should be captured electronically during routine care, as is done for neonatology [9].

For commissioning, WSIC enables examination of healthcare activity in segments of the population. This can support developing integrated services for individuals with similar needs and monitoring their outcomes. This functionality is under development as providers and commissioners move towards integrated care and start to define population outcomes. CCGs need a range of information, crucially including patient information. CCGs not only draw on evidence about what is most clinically or cost-effective but also consider patient experience and clinical staff’s local knowledge.

WSIC/Discover has been funded by NW London Collaboration of CCGs as well as Imperial College Healthcare Partners – a not-for-profit company owned by a partnership of NHS providers of healthcare services, CCGs and leading local universities. This initiative has been funded for 7 years by the funders and we are currently exploring the feasibility of the sustainability of this solution through licences. The fees for research access cover the administrative costs currently, but we would be moving more to a data licence fee to ensure sustainability. Any organisation wishing to follow this example would need to invest up-front to ensure the data asset and associated products are developed before licensing them: this will ensure a better buy-in from customers as the use cases will be met.

### Database technicalities

The WSIC database uses the Microsoft SQL Server 2012 Enterprise Edition platform and has a combined storage of approximately 1.5 TB. As commissioners are not legally permitted to view patient-level data, the data are provided by an intermediary service, the Data Services for Commissioners Regional Offices (DSCRO). Their task is to provide the acute, mental health and community activity data submitted by providers with clear patient identifiable information to the WSIC team, who carry out the data loading process and create the integrated care record through NHS Number linkage.

**Fig. 1:**
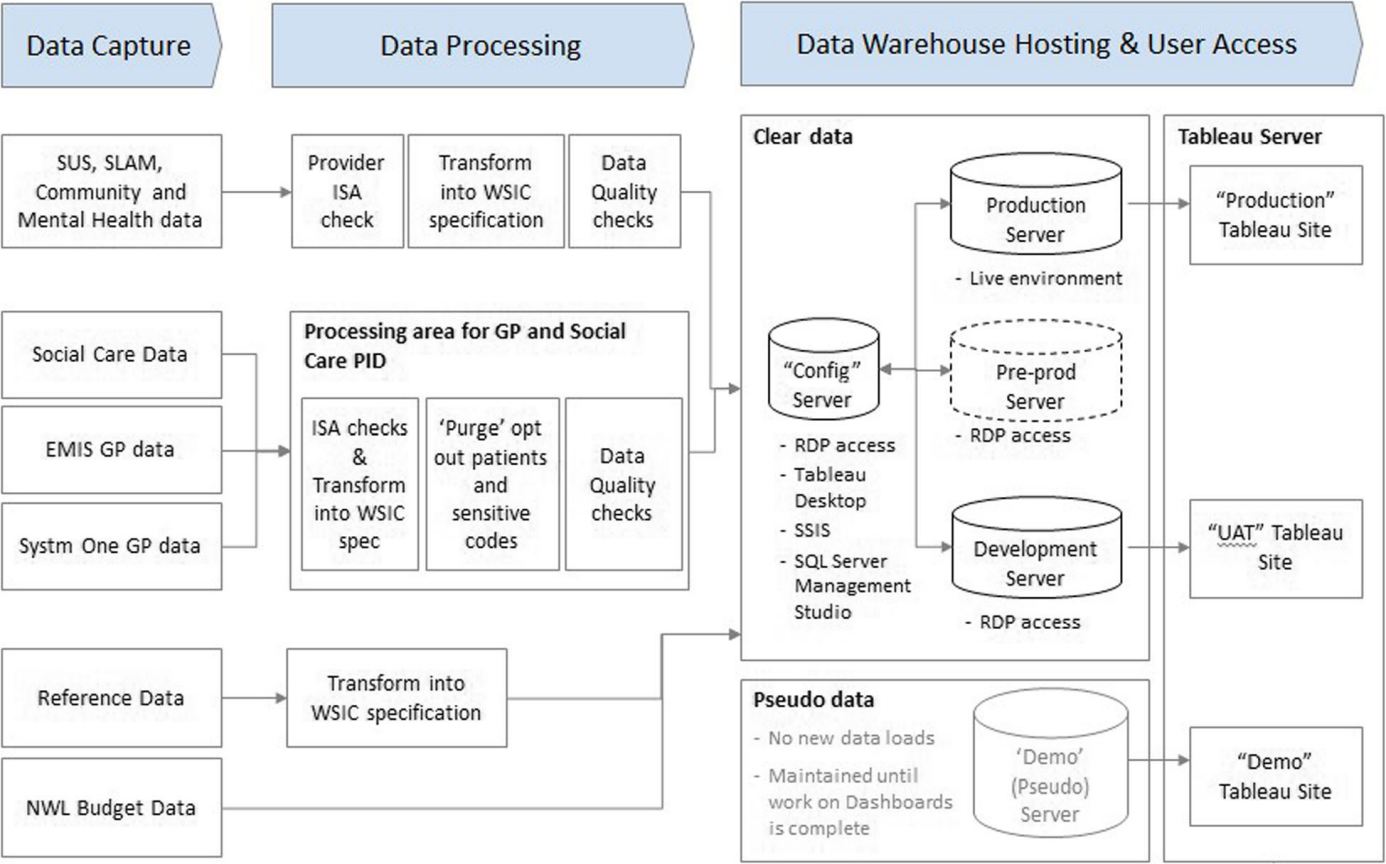
WSIC database architecture. SUS = Secondary User Service, SLAM = Service Level Agreement Management, EMIS = Egton Medical Information Systems, GP = General practitioner, NWL = North West London, WSIC = Whole System Integrated Care, ISA = Information Sharing Agreement, RDP = Remote desktop protocol, Tableau = Business Intelligence Software, SSIS = SQL Server Integration Services, SQL = Structured Query Language, UAT = User Acceptance Test

The primary care clinical systems SystmOne and EMIS are used in the WSIC area, from which data extraction company Apollo extracts the data directly. Apollo purge the sensitive codes (abortions etc) and patient opt-outs (patients who do not wish their records to be used except for direct care) and then pass the raw data files to the WSIC team. All the data are imported using the WSIC ETL (Extract, Transform, Load) layer, which is built from Microsoft’s Integration Services platform. The primary care data are processed in a separate ‘black box’ environment with restricted access and relevant security provisions to ensure that users are unable to view potentially sensitive data without permission. After ‘purging’, the data are transferred into the WSIC warehouse environment to be linked with secondary care and other data. The WSIC ETL layer contains error-handling features to ensure that invalid data are either redirected and removed from the reporting layer or logged and reported to the clinical users in the format of a Tableau dashboard while being imported to the reporting layer. Figure 1 shows the architecture.

A copy of the WSIC data is available in de-identified form that meets NHS data minimum standards. The version for service evaluation is stored on a dedicated server hosted by the Commissioning Support Unit. To gain access to the de-identified data set, a data access request form needs to be submitted by the Security and Access Subgroup for approval. Access is only provided for legitimate use by employees of an organisation that is a signatory of the NWL Digital Information Sharing Agreement (ISA); access may be sponsored by an ISA signatory. The data are provided as SQL tables.

Data held in WSIC are driven from an agreed data specification that has been signed off by the NWL Digital and Cyber Security Governance Group. This has been in operation since the development of the original Information Sharing Agreement (2015) and continues to meet monthly. Any changes to the WSIC data specification need to be approved by the NWL Governance Group.

### Accessing Discover for research

**Fig. 2:**
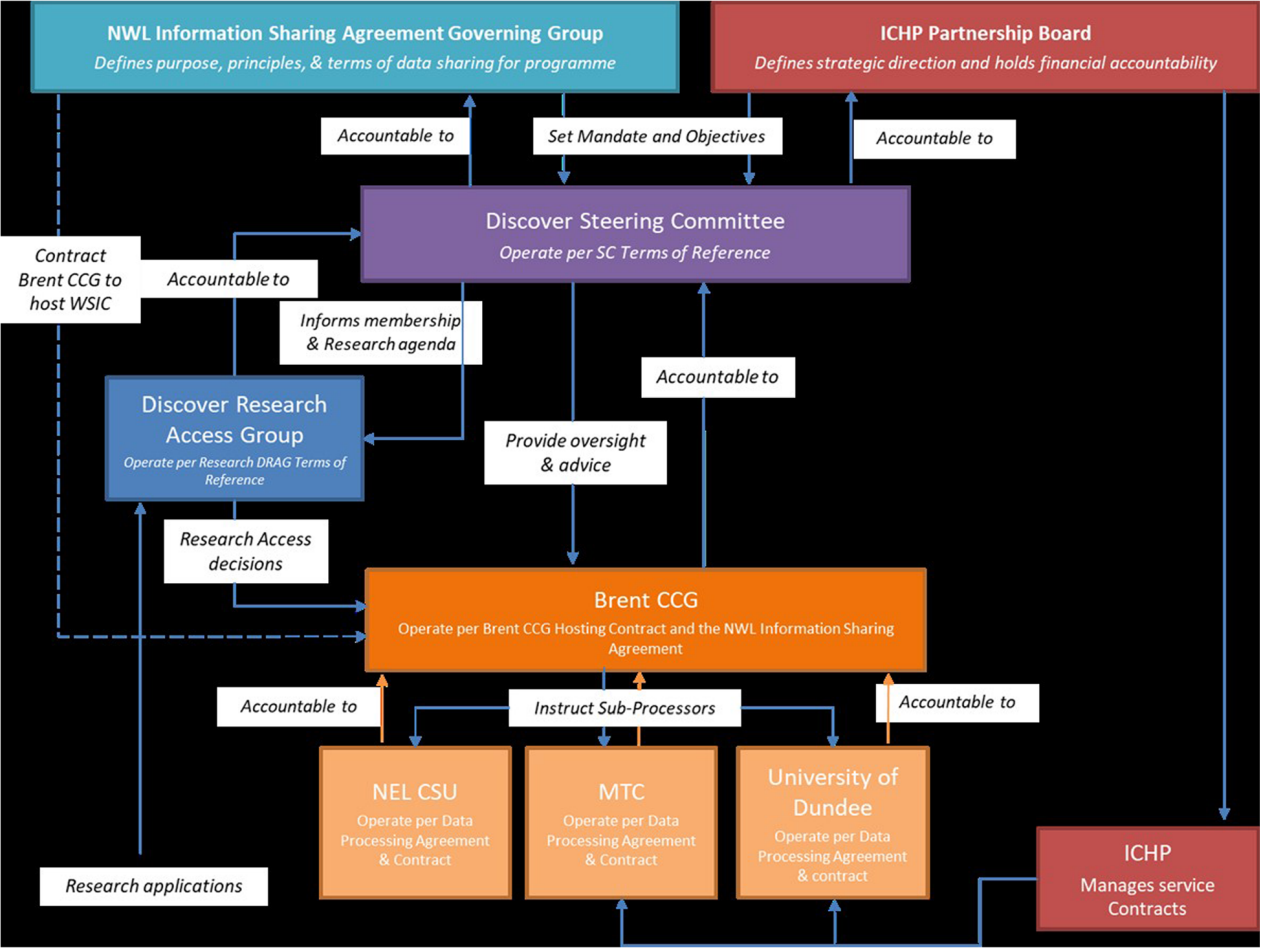
Discover Project Governance Framework

Researchers use the WSIC dataset on the platform set up by the Discover team. This use is managed by the governance structure in Fig. 2. The Discover Steering Group meets every 2 months, with broader membership coming from the R&D Directors from the Trusts, WSIC, the National Institute for Health Research, patient representatives and Imperial College Health Partners (ICHP). The Steering Group reports to both the ICHP Board and the NWL Digital Information Sharing Group. The purpose of the Steering Committee is to hold the Discover Data Access Group (DRAG) to account, informing wider stakeholder engagement and providing Discover with strategic direction and an executive decision-making function. The DRAG is chaired by a patient representative and meets monthly to review research proposals on Discover. It has responsibility for evaluating whether applications to access Discover are consistent with the Discover Principles Charter and that the requests do not pose undue risk to the individuals, communities or organisations to which they relate; this includes evaluation of risk of loss of privacy and assurance that appropriate protections of confidentiality and ethics review are in place. The Discover team has HRA approval for any retrospective studies submitted to the DRAG until 2023. See Appendix for details and links on how to access Discover.

### Consent-to-contact register

As well as retrospective studies with cross-sectional, time-series and cohort designs, WSIC can also be used for prospective follow-up studies including randomised controlled trials and cohort studies by tagging the electronic records of patients who have consented to take part. To do this, Discover is developing a register for people interested in contributing to health research. This includes anyone aged 18 and over living in NW London, either healthy people or those with a medical condition. This allows the Discover team to contact patients who are already consented to be contacted for research, speeding up recruitment. Launched in 2018, it has so far recruited over 3000 volunteers.

## Methods

Using descriptive statistics, we compared the Discover patient mix with that of London as a whole and the UK. Mid-year population estimates for London were taken from the London Trust [10] and for the UK were taken from the Office for National Statistics [11]. As Discover lacks the date of patient registration with the GP, its populations are currently only known on the day of data extraction, not historically. To obtain denominators for the proportion of patients with key risk factors recorded, the Discover population (denominator) over time was estimated based on a current comparison with London (see Appendix). Year-specific recording rates were calculated using the disease status as at Dec 31.

We estimated the prevalence of long-term conditions covered by the Quality and Outcomes Framework (QOF) programme for general practice; UK prevalence figures were taken from QOF for 2017/18 [12].

## Results

### Data elements and recording levels

**Fig. 3:**
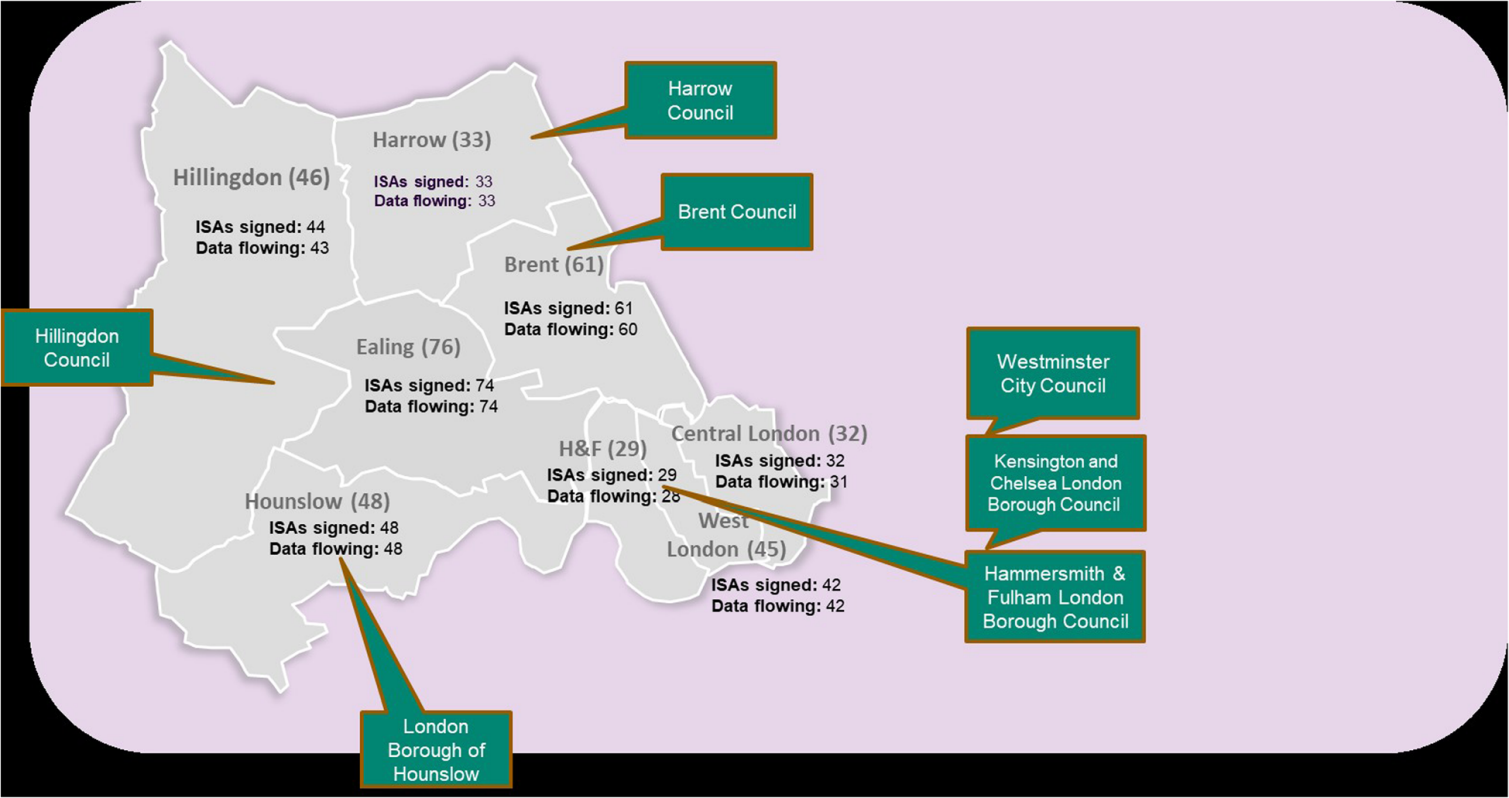
Geographical area of London covered by the WSIC database (ISA = information sharing agreement)

Figure 3 shows the geographical area of London covered by Discover and available for research. At June 11th 2019, it held records for a total of 2.37 M patients: the 365 participating general practices account for 95% of the total NW London population. Since Jan 1 2015, records include 334,463,392 primary care consultations, 5,186,708 ED visits, 20,038,402 outpatient appointments, 2,648,770 inpatient stays, 9,954,401 community activities, 20,668,088 mental health contacts and 379,409 number of records in the social care dataset. Table 1 describes the data elements’ level of aggregation and coding system.

**Table 1:** Discover data elements by level of aggregation

Data element	Event level	Patient level	Organisation level	Data coding system
Demographics		Y		n/a
GP or other primary care consultation	Y			Read codes
Clinical tests ordered in primary care	Y			Read codes
Referrals to secondary care	Y			Read codes
Practice staffing			Y	n/a
Social care contacts	Y			Unique to this data set
Community mental health	Y			Unique to this data set
Emergency department visits	Y			Unique to England
Hospital stays	Y			ICD10 for diagnoses; UK’s OPCS for procedures
Hospital outpatient appointments	Y			ICD10 for diagnoses; UK’s OPCS for procedures
Death registration		Y		ICD10 for causes
Geographical location (postcode)		Y	Y	UK postcode

**Fig. 4:**
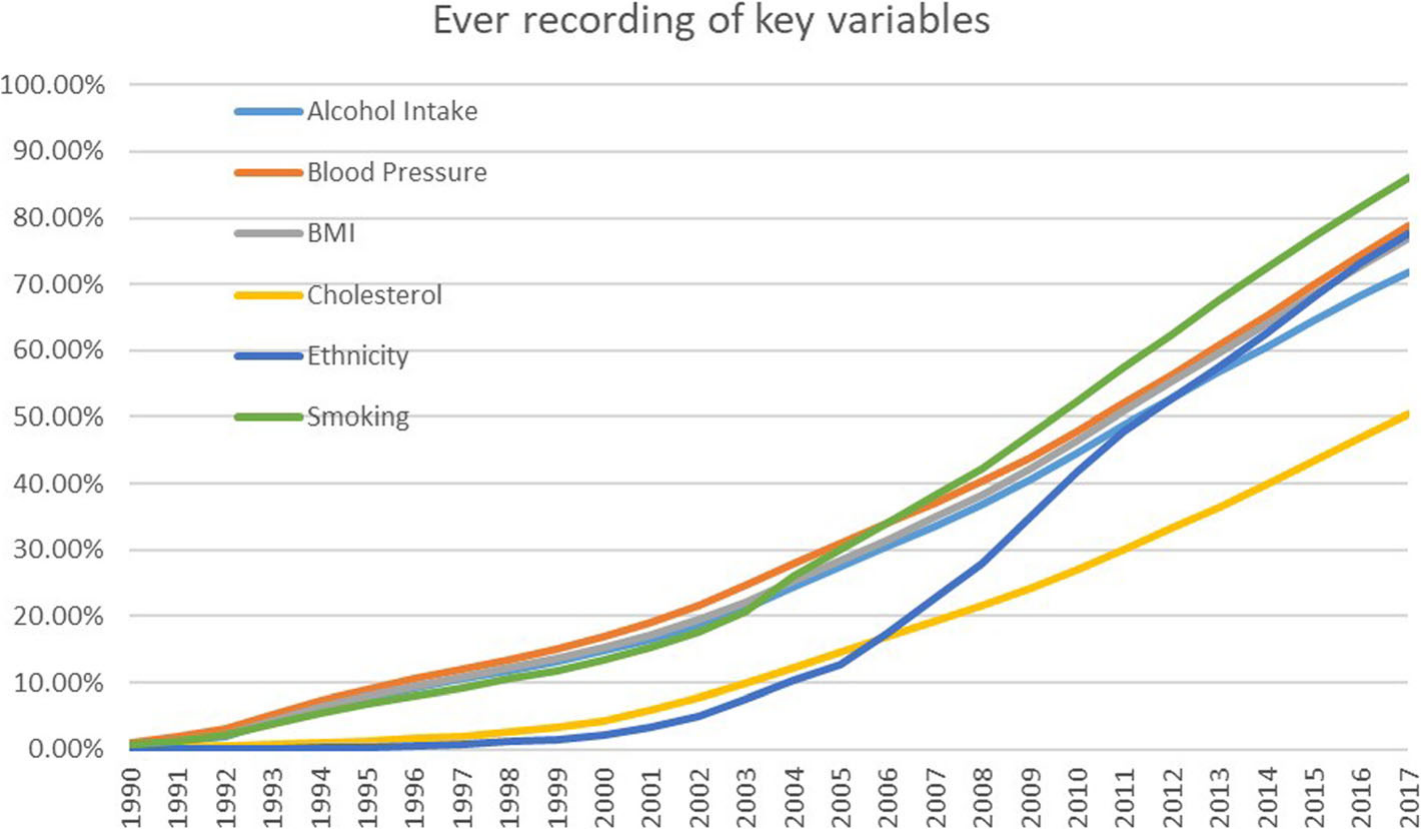
Recording levels over time for key risk factors

Even when sectors are included or data items exist in a given part of a component database, the completeness and accuracy of data items varies. Figure 4 shows how the recording completeness for six key risk factors has increased over time since its very low base in the 1990s. Recording levels in Discover are now above 70% for smoking, blood pressure, ethnicity, alcohol and BMI but not yet for cholesterol. As the underlying data are recorded by GPs in much the same way, the patterns in CPRD are similar [4].

#### Patient mix

**Fig. 5:**
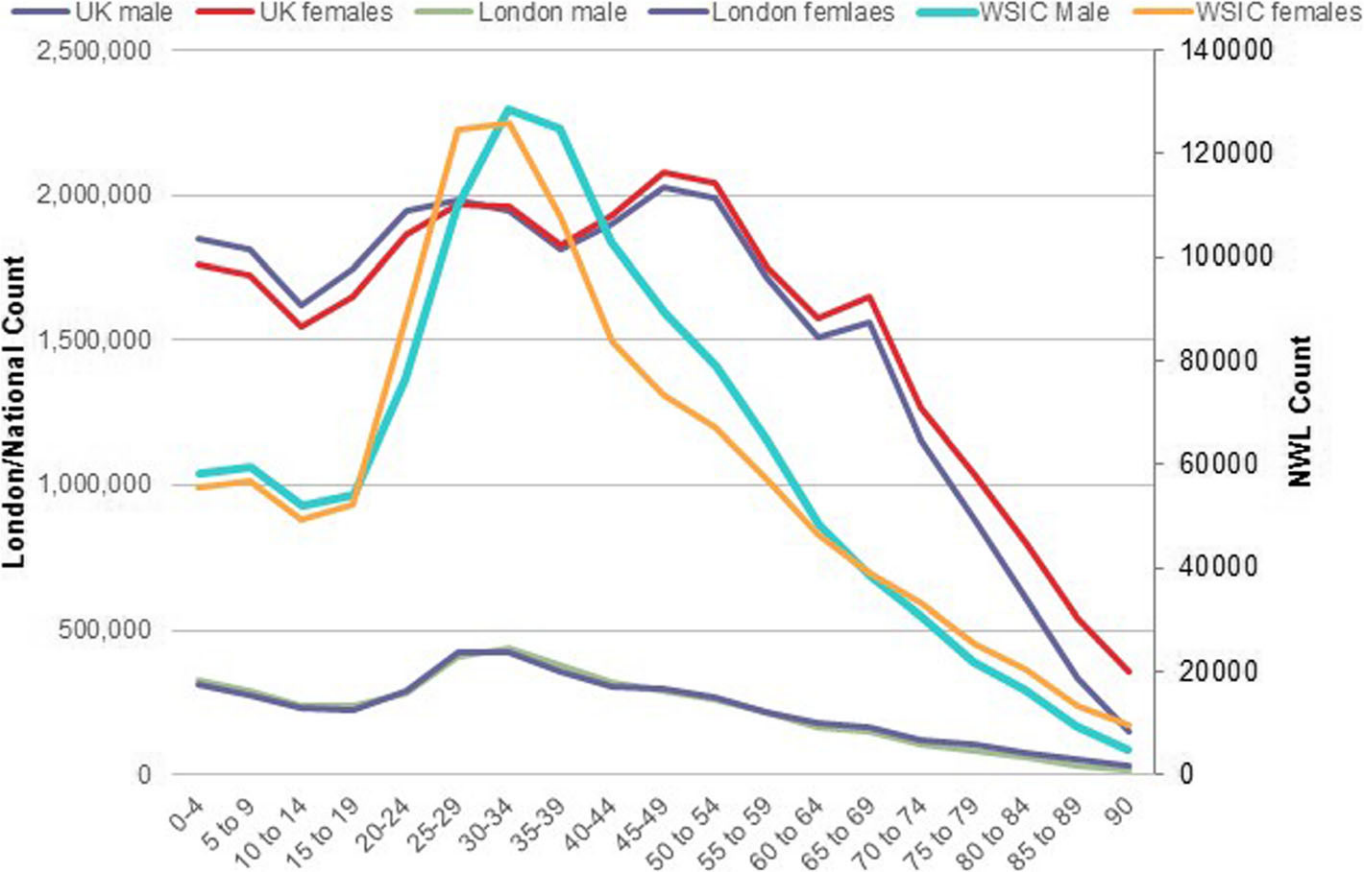
Comparison of age-gender mix of WSIC, London and UK populations (NWL = Northwest London). CCG = Clinical Commissioning Group, SC = Steering Committee, WSIC = Whole System Integrated Care, ICHP = Imperial College Health Partners, NEL = North East London, CSU = Commissioning Support Unit, MTC = Name of a company

Figure 5 shows close matches between the Discover population and both the overall London and national English age-gender distributions. However, the Discover population is more ethnically diverse, with 22% recorded as Asian or Asian British, 9% as mixed ethnicity, 6% as black or black British, 26% white, 36% unknown (most are likely to be white), and 1% other. The UK population as a whole is 87% white, 4% Asian or Asian British and 3% black or black British.

Table 2 gives the prevalence of diseases on the Quality and Outcomes Framework (QOF) list estimated in Discover, based on the most recent assessment of a patient before the calculation date. Most prevalence and incidence of the QOF diseases in the Discover population are comparable to the national rates.

**Table 2:** Prevalence estimates for long-term conditions covered by the Quality and Outcomes Framework for general practice

Condition	National published rate (2016/17)	WSIC / Discover (2019)
Asthma	5.9%	7.6%
Atrial Fibrillation	1.8%	1.0%
Cancer	2.0%	2.2%
Chronic Kidney Disease, age 18+	4.1%	1.6%
COPD	1.9%	2.2%
Coronary Heart Disease	3.2%	1.8%
Dementia	0.8%	0.4%
Depression & anxiety symptoms	9.1%	8.5%
Diabetes	6.7%	5.4%
Epilepsy, age 18+	1.0%	0.8%
Heart Failure	0.8%	0.7%
Hypertension	13.8%	9.5%
Learning Disability	0.3%	0.3%
Mental Health	0.9%	0.9%
Multiple sclerosis	164 per 100,000	127 per 100,000
Obesity, age 18+	9.7%	10.5%
Osteoporosis, age 50+	2.2%	3.7%
Peripheral Arterial Disease	0.6%	0.3%
Rheumatoid arthritis	0.7%	0.5%
Stroke and Transient Ischaemic Attack	1.8%	1.0%

### Comparison with CPRD

Table 3 contrasts Discover with CPRD by data element and time period covered. While the core primary-to-secondary linked components are common to both, Discover covers some extra sectors. Unlike in CPRD, institutions such as practices and hospitals are named in Discover, which, together with geographical identifiers (postcodes), allows maps to be created and network analysis, for example, to be applied to understand patient journeys. This allows for some analyses that are not usually possible in CPRD: for example, linkage with data on healthcare provider characteristics, such as staffing levels.

**Table 3:** Comparison between Discover and CPRD by data element and time period covered

Element	Discover	CPRD GOLD
Date of first capture of primary care consultations	Since 2000 (earlier data are available but of poorer accuracy)	Can be 1980s or earlier but depends on practice
Number of registered patients as of Nov 2018	2.3 million approx	11 million (active) approx. Across the UK
Number of participating GP practices as of Nov 2018	361 (out of 366 in the region)	718 in England [ref Kontopantelis 2018, referring to 2017], < 1200 in the UK
Number of participating practices linked to hospital data as of Nov 2018	361	411 in England (75% of participating English practices) [from CPRD website]
Linkage to hospital admissions	Y, all NWL-commissioned activity, inc to hospitals out of the region.	Y, nationally via HES*.
Linkage to ONS mortality data	N (forthcoming)	Y
Linkage to national clinical audits	N	Y. Bespoke and limited e.g. to MINAP; others in progress
Linkage to national registries	N	Y. National cancer registries and related treatment databases
*Type of information*		
Patient demographics	Y (only GP-registered pts)	Y (only GP-registered pts)
Prescribed medications	Y (GP-prescribed plus in-hospital high-cost drugs)	Y (GP-prescribed plus in-hospital high-cost drugs via HES linkage)
Social care data	Y	N
Community mental health data	Y	N (unless done within the GP practice)
Ambulance activity	N (coming soon)	N
Staffing	Y (e.g. practice and hospital)	N (can be requested by practice, but with some loss of information to preserve practice anonymity)
NHS 111	N (coming soon)	N
Healthcare costs as distinct from tariffs	Y (commissioner prices – see Appendix for details)	N
*Private care and other information*		
Private primary care	N**	N**
Private secondary care	N	N
Private social care	N	N
Wider determinants of health (crime, deprivation, pollution, education etc)	N, but area-level deprivation scores can be linked by user	Y: Index of Multiple Deprivation (IMD) linked to practice and patient via their postcode

*HES Hospital Episodes Statistics (national hospital administrative database for England)

**Private primary care is still only small-scale in the UK

Discover covers health and social care activity in NW London institutions, but national data for England are used for hospital admissions. This means that when a patient registered with a NW London GP accesses hospital care anywhere in England, this information is included in the warehouse. This is important because some patients are treated outside NW London, for example, in specialist hospitals further afield.

Like CPRD, Discover is gradually expanding its set of databases that are linked to its core offering. Some of this information is available in the national hospital administrative database used by both CPRD and Discover, but the recently added high-cost drugs database gives dosages and better breakdown by named drug rather than just drug class as in the hospital admissions data.

## Discussion

WSIC/Discover is one of a new breed of local but large linked databases, derived from health and social care records, used for service evaluation and commissioning and increasingly also for research. While smaller than CPRD in terms of the numbers of practices and patients, it offers advantages in the inclusion of social care and mental health data and identifiable general practices and providers, allowing easy incorporation of institution-level data for service evaluation and research.

To enable researchers to use data collected for non-research purposes, ethical issues around consent for secondary use of patient data, robust de-identification and information governance procedures have been established. While the relevant legislation will differ by country, even with the advent of Europe’s General Data Protection Regulation, these issues are relevant internationally. The development of a consent-to-contact register alongside the Discover dataset promises to make this powerful linked dataset a tool to run real-world studies retrospectively or prospectively.

Although the data have been de-identified, a secure platform and access controls are still needed due to the potential for re-identification, which is possible by linking the unencrypted parts of the record with known information about the individual. Public engagement has been crucial for the project to explain the risks and benefits, something that was done badly in England’s care.data initiative, designed to extract data from primary care medical records for commissioning and other purposes, including research [13].

There have been few published descriptions of local data warehouses like WSIC. One is the Kent Integrated Dataset [5]. As with CPRD and Discover, the primary care data in KID are the richest, but with all such data there are recording differences between general practices and over time. Among the many data elements that are captured, data quality for some remains variable. Symptom severity for COPD is captured quite well in primary care EHRs [14] but for other conditions this is not the case, and hospital records that use ICD10 for diagnoses will be of limited help. The 2004 introduction of the Quality and Outcomes Framework, in which some of the payments that GPs receive depends on their management of chronic conditions, helped drive improvements in recording of key risk factors and intermediate outcomes such as BP, smoking and HbA1c [15]. Similar future initiatives are likely to have a similar impact.

Like CPRD and other similar databases in the UK, WSIC has been several years in the making and is still growing with further linkages (Table 4).

**Table 4:** Potential future developments in WSIC

Data gap	System sector	Current state and potential development
Private hospitals	Hospital	No plans yet
Private care homes	Social care	No plans yet
Private GPs	Primary care	Still very small sector, but potential very limited
Inpatient medications	Hospital	High-cost drugs already captured, but other drugs will need to come from pharmacy databases
Inpatient scans	Hospital	National Diagnostic Imaging Data set is newest part of HES* and captures such tests but, crucially, not their results, which would come from other hospital-specific systems
Inpatient lab test results	Hospital	No plans yet
Quality of life	all	This can potentially be recorded using Read codes in GP records
Patient activation measure	all	Already captured for around 5000 patients, and the number is growing
Over the counter medication use	Community care	None unless reported by patient and coded by GP
Medication adherence by the patient	all	Some Read codes exist for chronic diseases in primary care, usage unknown; some published algorithms exist for use with CPRD to estimate this
Patient-reported outcome measures (PROMs)	all	Captured nationally only for 4 procedures, linked to HES*
Real healthcare cost rather than price to the payer	Primary and secondary care	Not yet
Ambulance service	ambulance	London Ambulance Service database to be linked soon
NHS 111 telephone advice service	n/a	In discussion
ONS mortality data	all	Not yet but high priority
National clinical audits and registries	all	Could be linked via NHS number; CPRD link to several national audits e.g. cancer registry

*HES Hospital Episodes Statistics (national hospital administrative database for England)

As well as the options considered in Table 4, future directions for databases like WSIC include the incorporation of data on patient-reported outcomes and telemonitoring data, for example entered by patients themselves via apps (several companies are doing this but generally only for patients with a given condition at enrolled practices) or collected from them via wearable sensors and analysed by machine learning. Other uses include long-term surveillance of medications, process mining and service redesign scenario modelling. There is government investment too: up to five Digital Innovation Hubs will be led by Health Data Research UK (HDR UK), the national institute for data science in health. Discover will be one of these, called “Discover-NOW”.

## Conclusions

WSIC/Discover has been several years in the making, with both similarities to and differences from CPRD. With the groundwork done, it is ready to expand yet further and, providing that users understand how the data were generated and the limitations, it is a valuable research tool and a model for others developing similar data warehouses.

## Supplementary information

**Additional file 1.**

## Data Availability

The WSIC / Discover data that support the findings of this study are available from Imperial College Partners, but restrictions apply to the availability of these data, which were used under licence for the current study, and so are not publicly available. Researchers wishing to access WSIC / Discover data can apply as described in the manuscript. National and London population estimates are available at URL: http://www.ons.gov.uk/ons/taxonomy/index.html?nscl=Population+Estimates (accessed Sep 6 2019, no DOI available). Quality and Outcomes Framework (QOF) prevalence data for 2016–17 are available at URL: https://digital.nhs.uk/data-and-information/publications/statistical/quality-and-outcomes-framework-achievement-prevalence-and-exceptions-data/quality-and-outcomes-framework-qof-2016-17 (accessed Sep 6 2019, no DOI available).

## Abbreviations

CCG: Clinical Commissioning Group
CPRD: Clinical Practice Research Datalink
DRAG: Discover Data Access Group
DSCRO: Data Services for Commissioners Regional Offices
ETL: Extract, Transform, Load
ICHP: Imperial College Health Partners
ISA: Information Sharing Agreement
NHS: National Health Service
QOF: Quality and Outcomes Framework
WSIC: Whole Systems Integrated Care
