# Supplementary material for: How an electronic health record became a real-world research resource: comparison between London’s Whole Systems Integrated Care database and the Clinical Practice Research Datalink
Source: BMC Med Inform Decis Mak. 2020 Apr 20;20:71. doi: 10.1186/s12911-020-1082-7 (PMC7171852; doi:10.1186/s12911-020-1082-7)
Supplement: Supplementary file 1 — Additional file 1. [file 12911_2020_1082_MOESM1_ESM.docx]

# Appendix for WSIC ms

**How to access Discover**

Discover is available to academic and NHS researchers in North West London. The researcher must either be employed or sponsored by an Information Sharing Agreement (ISA) signatory (please see list below).

If you are a commercial organisation and would like to utilise Discover services, you must be working in partnership with one of our local researchers. If you don’t have a local partner, we can work to facilitate this for you.

Step 1

Please download the relevant application form and submit to researchers@registerfordiscover.org.uk. You will need to submit ethical approval (where required) and R&D approval alongside your application.

Step 2

The Discover Team will review your application with you to ensure the requirements are possible and agree the analytical services required. A price for the service and a schedule for delivery will be provided (pending approval of the application).

Step 3

The application is then submitted to the monthly Discover Research Access Group for consideration and approval.

Step 4

Once approved, the Discover Team will deliver the request.

- North West London ISA Hospital Signatories

- Central and North West London NHS Foundation Trust

- London North West University Healthcare NHS Trust

- West London Mental Health NHS Trust

- Central London Community NHS Healthcare NHS Trust

- Chelsea and Westminster NHS Foundation Trust

- Imperial College Healthcare NHS Trust

- The Hillingdon Hospital NHS Foundation Trust

- The Royal Brompton Hospital Trust

The ISA also covers data from eight local authorities and most of the GP practices of North West London.

We have also developed Discover access guidelines that set out the application process for researchers who want to use Discover, including eligibility criteria and the application review checklist. These guidelines can be used to help inform your application to Discover.

Info on how to access to Discover is described using the following link:

<https://www.registerfordiscover.org.uk/researchers/how-to-access-discover>

**Calculating Patient Level Costs (taken from WSIC user guide)**

Patient level costs refer to the indicative spend calculated separately for each patient for each healthcare sector, as explained below.

GP Costs:

These are a combination of GMS/PMS/APMS contracts commissioned by NHS England and locally commissioned (CCG) schemes such as LIS/LES/OOHS.

Prescribing costs are not currently shown as they cannot be matched to individual patients. These costs reflect the actual Outturn costs for historic years and YTD budgets for the current year.

Practice level costs are apportioned across age groups based on historic analysis of appointment utilisation and then to patients, based on the number of recorded daily contacts that patient has had with the practice. The age group split is required as practices do not currently supply data for all registered patients e.g. children.

The cost allocation assumes that all patient contacts for the specified age group consumes the same resource so all contacts will have the same unit price.

Acute Costs (SLAM):

These are based on actual activity and costs (i.e. primarily cost per case) as reported by Trust issued patient level SLAM reports. Some contractual adjustments e.g. Emergency Threshold adjustments, Re-admission and other contractual penalties are applied retrospectively at the patient level.

Costs do not reflect the sometimes significant values that are not reported at patient level e.g. Direct Access or Contractual caps.

Community Costs:

These are based primarily on block contract values split across service lines or other groupings of activity.

Indicative activity plans are generally used to determine unit costs and values are assigned to the patient based on their share of the service line activity and include only those costs that can be traced back to a specific patient.

Mental Health Costs:

These are based primarily on block contracts, and therefore fixed in cost terms regardless of activity levels.

The values are assigned to patients based on the overall share of activity defined under In Scope services and Out of Scope services. In Scope services cover cluster bed days as defined under the national framework for mental health activity classification, whilst Out of Scope services cover locally defined services.

Social Care Costs:

These are based on the total cost of the various care packages received by the client. Clients can be receiving more than one care package at any one time and the costs only include those that have been specifically identified to the client less any contribution by the client.
